# The Impact of the COVID-19 Pandemic on the Care of Women Experiencing Abortion in a University Hospital in Brazil

**DOI:** 10.1055/s-0042-1759749

**Published:** 2023-04-27

**Authors:** Priscilla Brenda Fonseca Dantas, Carolina Braga Trabach, Aline Aparecida Junqueira, Carina Cordeiro Nunes, Nelio Neves Veiga-Junior, Luiz Francisco Baccaro

**Affiliations:** 1 Departamento de Tocoginecologia, Universidade Estadual de Campinas, Campinas, SP, Brazil

**Keywords:** CLAP MUSA network, perinatal information system, population surveillance, abortion, EviSIP, COVID-19, rede CLAP MUSA, sistema de informação perinatal, vigilância populacional, aborto, EviSIP, COVID-19

## Abstract

**Objective** To evaluate the impact of the coronavirus disease 2019 (COVID-19) pandemic on the care of patients with miscarriage and legal termination of pregnancy in a university hospital in Brazil.

**Methods** A cross-sectional study of women admitted for abortion due to any cause at Hospital da Mulher Prof. Dr. J. A. Pinotti of Universidade Estadual de Campinas (UNICAMP), Brazil, between July 2017 and September 2021. Dependent variables were abortion-related complications and legal interruption of pregnancy. Independent variables were prepandemic period (until February 2020) and pandemic period (from March 2020). The Cochran-Armitage test, Chi-squared test, Mann-Whitney test, and multiple logistic regression were used for statistical analysis.

**Results** Five-hundred sixty-one women were included, 376 during the prepandemic period and 185 in the pandemic period. Most patients during pandemic were single, without comorbidities, had unplanned pregnancy, and chose to initiate contraceptive method after hospital discharge. There was no significant tendency toward changes in the number of legal interruptions or complications. Complications were associated to failure of the contraceptive method (odds ratio [OR] 2.44; 95% confidence interval [CI] 1.23–4.84), gestational age (OR 1.126; 95% CI 1.039–1.219), and preparation of the uterine cervix with misoprostol (OR 1.99; 95% CI 1.01–3.96).

**Conclusion** There were no significant differences in duration of symptoms, transportation to the hospital, or tendency of reducing the number of legal abortions and increasing complications. The patients' profile probably reflects the impact of the pandemic on family planning.

## Resumo

**Objetivo** Avaliar o impacto da pandemia de *coronavirus disease 2019* (Covid-19) no atendimento de pacientes com aborto espontâneo e interrupção legal da gravidez em um hospital universitário no Brasil.

**Métodos** Estudo transversal com mulheres admitidas por aborto por qualquer causa no Hospital da Mulher Prof. Dr. J. A. Pinotti da Universidade de Campinas (UNICAMP), Brasil, entre julho de 2017 e setembro de 2021. As variáveis dependentes foram complicações relacionadas ao aborto e interrupção legal da gravidez. As variáveis independentes foram período pré-pandemia (até fevereiro de 2020) e período pandêmico (a partir de março de 2020). O teste de Cochran-Armitage, teste do qui-quadrado, teste de Mann-Whitney e regressão logística múltipla foram utilizados para análise estatística.

**Resultados** Foram incluídas 561 mulheres, 376 no período pré-pandemia e 185 no período pandêmico. A maioria das pacientes durante a pandemia era solteira, sem comorbidades, teve gravidez não planejada e optou por iniciar método anticoncepcional após a alta hospitalar. Não houve tendência significativa para mudanças no número de interrupções legais ou complicações. As complicações foram associadas a: falha do método contraceptivo (razão de chances [RC] 2,44; intervalo de confiança [IC] 95% 1,23–4,84), idade gestacional (RC 1,126; IC 95% 1,039–1,219) e preparo do colo uterino com misoprostol (RC 1,99; IC 95% 1,01–3,96).

**Conclusão** Não houve diferenças significativas na duração dos sintomas, transporte ao hospital ou tendência de redução do número de abortos legais e aumento de complicações. O perfil das pacientes provavelmente reflete o impacto da pandemia no planejamento familiar.

## Introduction

The coronavirus disease 2019 (COVID-19) pandemic has affected health services around the world, requiring prioritization of the professional team and hospitals to meet the growing demand for cases complicated by the infection. Thus, the health system was structured in such a way to care for infected patients, limiting their surgical care to emergency procedures, leaving their surgical centers available to be transformed into intensive units and saving personal protective equipment. 1 Likewise, the general population was affected, losing track of its chronic diseases and measures to promote health and prevent illness and injury. Mandatory home isolation was instituted in several countries, and people were advised to seek medical attention only in urgent and emergency cases. Given these facts, these changes affected when patients seek and how they receive their medical care. 2

In relation to women in abortion situations, they had greater difficulty in accessing health services, since the basic health networks restricted their care; public transport reduced its fleet; the fear and the need for home isolation left the population without adequate screening or isolated until the appearance of alarm signals, delaying their care, diagnosis, and proper management, just as hospitals reduced the number of available beds, reserving hospitalization only for more critical cases, among other various socioeconomic and political factors that have impacted our current global health condition. 3

With this in mind, in March 2020, the American College of Surgeons (ACS) recommended delay of all nonessential invasive procedures, reinforcing, however, the importance of not delaying gynecologic emergency procedures, including ectopic pregnancy and miscarriage. 4 Likewise, the American College of Obstetricians and Gynecologists (ACOG) stated that care for women in abortion situations should be guaranteed by community- and hospital-based clinicians, as sometimes a delay of weeks or days may increase the risks or potentially make it completely inaccessible. 5

The COVID-19 pandemic triggered several changes in the flow of care for women experiencing an abortion. In the context outside the institution, we supposed that the reduction in the number of consultations available in basic health units and the reduction in the availability of public transport would delay the care, diagnosis, and assistance of these women, resulting in longer time experiencing symptoms and getting to the hospital. In the internal context of the institution, we can mention a reduction in the number of spaces available for hospitalization due to the need for distance between beds, and the reduction in the availability of intensive care unit (ICU) beds could limit the access of these patients to tertiary assistance.

This scenario raises the following research question: What influence did the changes in routine resulting from the COVID-19 pandemic have on the quality of care for women experiencing abortion in a university hospital? The aim of this study was to evaluate the impact of the COVID-19 pandemic on the care of patients with miscarriage and legal termination of pregnancy in a university hospital in Brazil.

## Methods

The multicentric network MUSA—Women in Abortion Situations—is a network created by the Latin American Center for Perinatology (CLAP, in the Portuguese acronym) to improve care for women undergoing any kind of pregnancy loss during the first half of pregnancy (spontaneous or induced ones) in Latin America and the Caribbean. 6 It includes several hospitals, called sentinel centers, which periodically send their data regarding the pregnancy cycle for registration in the Perinatal Computerized System (SIP, in the Portuguese acronym), a software developed by CLAP that helps health facilities register data related to pregnancy and epidemiologic monitoring. Our institution, University of Campinas Women's Hospital (UNICAMP) is a tertiary referral hospital for cases of complications related to pregnancy in municipalities in the region and experiences an average of 250 births and 20 cases of first trimester pregnancy loss per month. Our hospital has been a sentinel institution of the MUSA network since July 2017, prospectively collecting data which have already been used in other cross-sectional studies. However, this is the first work performed during the COVID-19 pandemic. The hospital follows the laws of Brazil regarding the legal termination of pregnancy, in which abortion is allowed only in cases of risk of maternal death, sexual violence, and fetal anencephaly. 7 8 9 10 11 12 13 14 15 16 17 18 19

The sentinel centers of the MUSA network regularly provide information on maternal morbidity in early pregnancy loss, termination methods for uterine evacuations, incidence of complications related to pregnancy termination, incidence of preoperative antibiotic use and prescription of contraception before hospital discharge. Through SIP, it is possible to carry out epidemiological monitoring and comparisons between different sentinel centers over time. Representatives from each sentinel center also hold regular online meetings to discuss the data collected, conduct scientific discussions on the topic of women's health in abortion situations, and encourage good clinical practices for safe abortion.

This cross-sectional study with epidemiological surveillance data was conducted between July 2017 and September 2021. All cases from the SIP-abortion database from July 1 ^st^ , 2017, to September 30 ^th^ , 2021, were included. The inclusion criteria were women admitted for spontaneous pregnancy loss (inevitable miscarriage, complete, incomplete, or missed abortions) and legal interruption of pregnancy due to any cause or any age group who visited our hospital. The exclusion criteria were women with bleeding during pregnancy who did not have a confirmed abortion and women with ectopic or molar pregnancies. The research ethics committee of our institute approved this study (approval number CAAE: 56933116.0.1001.5404).

The dependent variables evaluated were: abortion-related complications (infection, excessive bleeding, and intraoperative complications, such as postspinal anesthesia headache, disseminated intravascular coagulation, reapproach, and allergic reaction) and legal interruption of pregnancy.

The independent variables were Pre-pandemic period (PrP): from July 1 ^st^ , 2017, until February 29 ^th^ , 2020; and pandemic period (PP): from March 1 ^st^ , 2020, to September 30 ^th^ , 2021.

The control variables were patients' clinical and sociodemographic characteristics, such as age, education, marital status, living status, health records, number of pregnancies, number of births, number of abortions, body mass index (BMI), active smoking, illegal drug use, alcohol use, planned pregnancy, pregnancy resulting from contraceptive failure, date of admission at the hospital, if it is a medically induced abortion for legal reasons, gestational age, presence of any complications, and admission data, besides duration of transportation and symptoms.

Initially, a descriptive analysis of the data was performed. For continuous variables, the mean, standard deviation, median, minimum, maximum, and quartiles were calculated. For categorical variables, the relative frequencies were calculated. To assess whether there was a change in the trend in the occurrence of the outcome variables, the Cochran-Armitage trend test was performed. To evaluate the association between abortion-related complications and the independent variables, the Chi-squared or Fisher exact tests were performed for categorical variables, and the Mann-Whitney or Kruskal-Wallis tests for continuous variables. To evaluate the factors independently associated with abortion-related complications, a multiple logistic regression was performed, with “stepwise” selection criteria for variables. The significance level assumed was 5%. The software used for the analyses was the SAS System for Windows, version 9.2. (SAS Institute Inc., Cary, NC, USA).

## Results

During the study period, 561 women in a situation of abortion were included; 376 during the PrP and 185 during the PP. From the PrP, 50 women had abortion induced for legal reasons and 326 had other types of abortion. During the PP, 20 patients had legal abortions, and 165 had other types. During the PrP, it was observed that the mean maternal age was 30.13 ± 7.55 years, while in the PP, it was 30.21 ± 7.55 years. The mean gestational age was 11.03 ± 3.56 weeks in the PrP and 11.39 ± 3.53 weeks in the PP. The mean number of previous births was 1.18 ± 1.22 births in the PrP and 1.41 ± 1.19 in the PP ( *p* = 0.014). The mean body mass index (BMI) was 27.25 ± 5.90 in the PrP and 26.16 ± 6.09 in the PP ( *p* = 0.014). The duration of symptoms was 3.59 ± 7.80 days, and the duration of transportation was 53.16 ± 125.57 minutes in the PrP, while in the PP, it was 4.30 ± 7.99 days and 33.94 ± 18.81 minutes, respectively ( Table 1 ).

**Table 1:** Clinical and sociodemographic characteristics of women in abortion situations - quantitative variables ( *n* = 561)

Period	Variable	Mean	SD	Median	Min	Max	*P* -value*
**Pre-pandemic**	Age ^a^	30.13	7.55	30.0	12.0	48.0	0.806
	Births ^b^	1.18	1.22	1.0	0	7.00	0.014
	BMI ^c^	27.25	5.90	26.45	15.60	45.34	0.014
	Gestational age ^d^	11.03	3.56	10.43	2.14	24.71	0.232
	Duration of symptoms ^e^	3.59	7.80	1.00	0	90.00	0.872
	Duration of transportation ^f^	53.16	125.57	30.00	0	1800.0	0.136
**Pandemic**	Age	30.21	7.55	30.0	11.0	46.0	
	Births ^g^	1.41	1.19	1.00	0	5.00	
	BMI ^h^	26.16	6.09	24.65	14.57	51.86	
	Gestational age ^i^	11.39	3.53	10.71	5.00	23.43	
	Duration of symptoms ^j^	4.30	7.99	1.00	0	40.00	
	Duration of transportation ^k^	33.94	18.81	30.00	5.00	40.00	

Abbreviations: BMI, body mass index; Min, minimum; Max, maximum; SD, standard deviation.

Missing data: a = 1; b = 19; c = 27; d = 5; e = 13; f = 6; g = 23; h = 4; i = 7; j = 18; k = 19.

* *P* -value referring to the Mann-Whitney test for comparing values between pre pandemic and pandemic group.

In the PrP, 60.93% of the patients were married or living in a stable relationship, while 51.65% in the PP were single ( *p* = 0.005). In the PrP, 91.96% of patients did not have comorbidities, compared with 96.74% in the PP ( *p* = 0.031). In the PrP, 12.1% of patients declared drinking alcohol, while only 5.41% did in the PP ( *p* = 0.013). A total of 32.71% of pregnancies were planned during the PrP, whereas 24.32% were had been planned in the PP ( *p* = 0.042). In the PrP, most patients (42.36%) were accompanied by their partners, while in the PP, most patients (45.36%) came alone ( *p* = 0.012). In the PrP, most patients (62.73%) chose not to initiate contraceptive methods at hospital discharge, while in the PP, 53.01% chose to initiate ( *p* < 0.001). In the PrP, most uterine-emptying procedures involved medication plus uterine curettage (41.49%), while in the PP, 40.44% underwent manual intrauterine aspiration ( *p* < 0.001) ( Table 2 ).

**Table 2:** Clinical and sociodemographic characteristics of women in abortion situations - categorical variables ( *n* = 561)

	PrP n	PrP %	PP n	PP%	*P* -value*
Age ^a^					
< 20 years	24	6.40	12	6.49	0.988
20–29 years	160	42.67	79	42.70	
30–39 years	144	38.40	69	37.30	
40–49 years	47	12.53	25	13.51	
Marital status ^b^					
Married/cohabiting	223	60.93	88	48.35	0.005
Single/other	143	39.07	94	51.65	
Comorbidities ^c^					
No	343	91.96	178	96.74	0.031
Yes	30	8.04	6	3.26	
Alcohol intake					
No	327	87.90	175	94.59	0.013
Yes	45	12.10	10	5.41	
Planned pregnancy ^e^					
No	251	67.29	140	75.68	0.042
Yes	122	32.71	45	24.32	
Companion ^f^					
Partner	158	42.36	53	28.96	0.012
Family	64	17.16	31	16.94	
Other	23	6.17	16	8.74	
None	128	34.32	83	45.36	
Contraception ^g^					
No	234	62.73	86	46.99	< 0.001
Yes	139	37.27	97	53.01	
Uterine emptying procedures ^h^					
None	34	9.04	13	7.10	< 0.001
MVA	0	0.00	74	40.44	
VA	0	0.00	1	0.55	
CTG	146	38.83	69	37.7	
MED	40	10.64	16	8.74	
CTG/MED	156	41.49	10	5.46	

Abbreviations: CTG, curettage; MED, medicated; MVA, manual vacuum aspiration; PP, pandemic period; PrP, pre-pandemic period; VA, vacuum aspiration.

Missing data: a = 1; b = 13; c = 4; d = 4; e = 3; f = 5; g = 5, h = 3. *p-value referring to the Chi-squared test for comparing values between pre pandemic and pandemic group.

Missing data: a = 11; b = 2; c = 2.

**Fig. 1:**
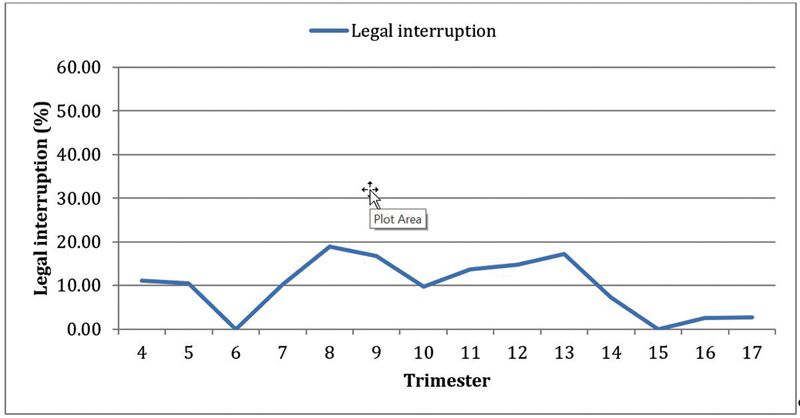
Number of legal interruptions of pregnancy between July 2017 and September 2021 by trimestral period. Cochran-Armitage test: Z = -0.28; *p* = 0.783.

Since the beginning of the evaluation period, 70 women (12.47%) had undergone legal interruption. We did not observe a significant tendency toward an increase or decrease in the number of legal interruptions. (Cochran-Armitage test: Z = -0.28; *p* = 0.783) ( Fig. 1 ).

**Fig. 2:**
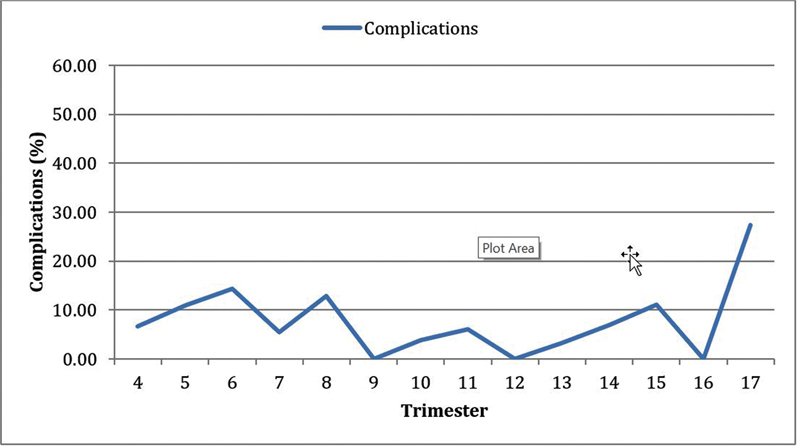
Number of complications associated with abortion between July 2017 and September 2021 by trimestral period. Cochran-Armitage test: Z = 0.05; *p* = 0.960.

Since the beginning of the evaluation period, 31 women (5.53%) had abortion-related complications. Among the complications, we found that the most frequents were: infection, with 13 cases (2.32%), and 8 cases of sepsis (1.43%); excessive bleeding, with 9 cases (1.60%), and 2 cases of hypovolemic shock (0.36%); and other complications, with 6 cases (1.07%), which include post-spinal anesthesia headache, disseminated intravascular coagulation, reapproach, and allergic reaction. We did not observe a significant tendency toward an increase or decrease in the number of complications. (Cochran-Armitage test: Z = 0.05; *p* = 0.960) ( Fig. 2 ).

After analyzing the factors associated with a higher prevalence of complications, considering the PP and PrP as independent variables, we observed that the pandemic period was not associated with a higher occurrence of complications. We observed that the factors associated with the occurrence of complications were: failure of contraceptive method ( *p* = 0.002); no cervical preparation with misoprostol ( *p* = 0.006); type of procedure performed for uterine evacuation, with curettage being the method with the highest number of complications ( *p* = 0.009); maternal age, with a higher number of complications among younger patients ( *p* = 0.031); gestational age, with more complications in more advanced pregnancies ( *p* = 0.010); and duration of symptoms, with more complications associated with longer duration ( *p* = 0.045) ( Tables 3 and 4 ).

**Table 3:** Factors associated with complications - categorical variables ( *n* = 561)

Variables	Complications		*P* -value
Contraceptive ^a^	No (n/%)	Yes (n/%)	*p* = 0.002
No	399/77.93	21/55.26	
Yes	113/22.07	17/44.74	
Misoprostol use ^b^			
No	203/39.04	24/61.54	*p* = 0.006
Yes	317/60.96	15/38.46	
Uterine evacuation ^c^			
None	45/8.65	2/5.13	*p* = 0.009
MVA	68/13.08	6/15.38	
VA	0/0	1/2.56	
CTG	194/37.31	21/53.85	
MED	56/10.77	0	
CTG/MED	157/30.19	9/23.08	

Abbreviations: CTG, curettage; MED, medicated; MVA, manual vacuum aspiration; VA, vacuum aspiration.

Missing data: a = 11; b = 2; c = 2.

**Table 4:** Factors associated with complications - quantitative variables ( *n* = 561)

Complication	Variable	Mean	SD	Median	Min	Max	*P* -value
**No**	Age ^a^	30.34	7.59	30.0	11.0	48.0	**0.031**
	Gestational age ^b^	11.01	3.44	10.43	8.43	24.71	**0.010**
	Duration of symptoms ^c^	3.51	6.69	1.00	0	60.00	**0.045**
**Yes**	Age	27.69	6.52	26.0	16.0	41.0	
	Gestational age ^d^	13.03	4.54	14.00	4.00	20.29	
	Duration of symptoms ^e^	7.77	16.47	2.00	0	90.00	

Abbreviations: SD, standard deviation; Min, minimum; Max, maximum.

Missing data: a = 1; b = 9; c = 30; d = 3; e = 1.

In the multiple logistic regression model, it was found that the variables significantly related to complications were: failure of the contraceptive method, with a risk 2.4 times greater (odds ratio [OR] 2.44; 95% confidence interval [CI] 1.23–4.84); gestational age, with an increase of 12.6% for every 1 week of gestational age (OR 1.126; 95% CI 1.039–1.219); and lack of uterine cervix preparation with misoprostol, raising 2.0 times the risk of complications (OR 1.99; 95% CI 1.01–3.96) ( Table 5 ).

**Table 5:** Factors associated with complications - Multiple logistic regression ( *n* = 503)

Variables	Categories	OR	OR (95% CI)	*P* -value
Contraceptive	No (ref.)	1.00	−	−
	Yes	2.77	1.34–5.74	0.006
Gestational age	Continuous variable (weeks)	1.149	1.051–1.256	0.002
Misoprostol use	Yes (ref.)	1.00	−	−
	No	2.18	1.05–4.51	0.037

Abbreviations: CI, confidence interval; OR, odds ratio; ref, reference.

No complications: *n* = 469; complications: *n* = 34.

Cases with missing variables were not included in the multiple analysis. Stepwise criteria for variable selection.

## Discussion

The COVID-19 pandemic has affected health services around the world and has changed how the general population experienced their diseases and sought health assistance. In relation to women in abortion situations, we supposed that changes outside the institution could hinder assistance of these women, and changes inside the institution could limit their access to the hospital. These facts brought us to the importance of evaluating the impact of the COVID-19 pandemic on the care of patients with miscarriages and legal termination of pregnancy in a university hospital in Brazil.

Although we have experienced these external changes, we did not observe great differences in the duration of symptoms and time of transportation to our hospital. We imagine that, as most primary care services were closed or turned to care for patients suspected of having COVID-19, the patients probably sought our emergency room as a first form of care, as well as assuming that access to public transport was guaranteed in our city and region of coverage. However, this is not what we expected, as facing COVID-19 pandemic changes in transportation contributed to increase health disparities, hindering access to healthcare to low-income families. 8

Regarding demographic aspects, we found that, during the pandemic, most patients were single, without comorbidities, experiencing abortions as a result of unplanned pregnancy and chose to start contraceptive methods at hospital discharge. These findings make us reflect on how the pandemic may have impaired family planning and access to contraceptive methods. We have learned from previous public health emergencies, such as the Ebola outbreak, that the impact of an epidemic on sexual and reproductive health is not a direct consequence of the infection, but an indirect result from strained health care systems, disruptions in care and redirected resources. 9 Riley et al. 9 estimated that a decline of 10% in the use of short- and long-acting reversible contraceptive methods in low- and middle-income countries due to reduced access would result in 49 million women without family planning support and 15 million unintended pregnancies over a year.

We observed an increasing in the number of women who were hospitalized without companions. It might be influenced by the internal restructuring of our service, since it restricted the number of companions and hospital visits during the pandemic period. However, our hospital guaranteed and prioritized the presence of companions for adolescents, victims of sexual violence, and women with important physical and emotional needs.

A new tendency was also observed in our hospital. Most uterine evacuation procedures performed during the pandemic were manual vacuum aspirations (MVAs), comparing to the previous tendency of using medicine for cervix preparation and curettage. Since the implementation of the MUSA network in our hospital, it was possible to generate data to assess our trends in clinical practice patterns, and the data were necessary for analyses of the safety of abortion practices and to purpose improvements in the quality of patient care and overall health outcome. In 2020, we began an intense process to insert MVA into our care practice, training the technical team, as well as modifying the institutional protocol and making MVA a priority method of uterine evacuation for abortions up to 12 weeks of gestational age, following the recommendation of the World Health Organization (WHO) and the Federation of Gynecology and Obstetrics (FIGO). 10 The adherence to the implementation was probably facilitated by the period of the pandemic, since it is a quick and easy procedure, with a lower risk of complications; it requires less complex anesthetic procedures and has a rapid recovery, allowing early hospital discharge. 10 11

We feared that external changes in health services organization and in people behavior during the pandemic could restrict women access to our hospital, resulting in a decrease of number of legal terminations of pregnancy and an increase in abortion-related complications. However, we did not observe this tendency, corroborating the hypothesis previously mentioned that patients sought for emergency attendance after primary care and that the access to our hospital was maintained during the pandemic period. This result differs from those of national data, which showed that only 55% of the 76 hospitals in our country that provide legal abortions were operating in 2019. 12

Our multivariate analysis showed that the variables significantly related to complications were failure of the contraceptive method, higher gestational age, and no preparation of the uterine cervix with misoprostol.

It is known that women using contraceptive methods can possibly not recognize symptoms of pregnancy, resulting in late diagnosis and delay in seeking medical attention, increasing the risk of infection and/or hemorrhage. 13 Also, because of the unplanned pregnancy, women need to face some unexpected issues, such as finding transportation or companion and justifying absence at work. Fear, embarrassment, or stigma are also barriers to seek care. 16 17 Besides, failure of contraceptive methods might reflect the contraceptive use pattern of our country, in which oral contraceptives and condom are predominant compared with long-acting reversible contraceptives, such as intrauterine devices. 13

Our study showed that each week of gestational age increased 12.6% the risk of complications, while another study showed an increase in the number of complications by up to 20% in each week. 18 Pregnancies with higher gestational ages mean higher uterine volume, bigger amount of retained products of conception, and possible chorioamnionitis. 13 The main complications include uterine perforation, cervical laceration, hemorrhage, uterine rupture, and infection. 14

Preparing the cervix prior to the procedure reduces this risk to less than 1% of cases. 15 Compared with manual dilation alone, it improves cervical dilation, shortens procedure times and decreases the risk of complications intraoperatively, such as cervical laceration and uterine perforation. 14

This study had some limitations. First, it was a cross-sectional study; thus, a cause-effect relationship could not be established. Furthermore, it was not possible to differentiate provoked abortion from spontaneous abortion, except in cases of legal induction. However, our study was important to evaluate the impact of the COVID-19 pandemic, which caused changes all over the world and could impact negatively in women's experiencing abortion.

We were pleased to find that our patients apparently did not have difficulties accessing our health service, mostly because we maintained and prioritized care for women in abortion situations despite all the reorganization and limitation we have suffered internally in the context of the pandemic.

## Conclusion

Our service did not reduce its volume of abortion attendance during the COVID-19 pandemic. Significant differences in the duration of symptoms and transportation to the hospital were not observed, neither was there a tendency to reduce the number of legal abortions, or an increase in complications. Despite reorganization of hospital function due to this public health emergency, we were one of 55% of services still providing legal abortions in our country. Our patients' profiles reflect the impact of the pandemic on sexual and reproductive health. This outbreak situation showed us that, in our institution, the infection might not have directly affected how women have experienced abortion, but how the reorganization of health system impacted on family planning.
